# Prognostic factors in localized Ewing's tumours and peripheral neuroectodermal tumours: the third study of the French Society of Paediatric Oncology (EW88 study)

**DOI:** 10.1054/bjoc.2001.2150

**Published:** 2001-11

**Authors:** O Oberlin, M C Le Deley, B N'Guyen Bui, J C Gentet, T Philip, P Terrier, C Carrie, F Mechinaud, C Schmitt, A Babin-Boillettot, J Michon

**Affiliations:** Pédiatrie Institut Gustave Roussy, 39 rue Camille Desmoulins, Villejuif Cedex, 94805; Département de statistiques Institut Gustave Roussy, 39 rue Camille Desmoulins, Villejuif Cedex, 94805; Oncologie, Fondation Bergonié, 180 Route de Saint Genès, Bordeaux Cedex, 33076; Oncologie, Hopital d'Enfants de La Timone, Avenue Jean Moulin, Marseille Cedex, 13385; Pédiatrie Centre Léon Bérard, 28 rue Laennec, Lyon Cedex 08, 69008; Anatomie Pathologique Institut Gustave Roussy, 39 rue Camille Desmoulins, Villejuif Cedex, 94805; Radiothérapie, Centre Léon Bérard, 28 rue Laennec, Lyon Cedex 08, 69373; Oncologie Pédiatrique CHR, Quai Moncousu, Nantes Cedex, 44035; Médecine Infantile Hopital d'Enfants, Rue du Morvan, Vandoeuvre Cedex, 54511; Onco-Hématologie, Hopital de Hautepierre, Avenue Molière, Strasbourg Cedex, 67098; Pédiatrie Institut Curie, 26 rue d'Ulm, Paris Cedex 05, 75231, France

**Keywords:** Ewing's tumour, chemotherapy, prognostic factors

## Abstract

*Purpose*: (1) To improve survival rates in patients with Ewing's sarcoma (ES) or peripheral neuroectodermal tumours (PNET) using semi-continuous chemotherapy and aiming to peform surgery in all; (2) To identify early prognostic factors to tailor therapy for future studies. *Patients and methods* One hundred and forty-one patients were entered onto the trial between January 1988 and December 1991. Induction therapy consisted of five courses of Cytoxan, 150 mg/m^2^ × 7 days, followed by Doxorubicin, 35 mg/m^2^ i.v on day 8 given at short intervals. Surgery was recommended whenever possible. The delivery of radiation therapy was based on the quality of resection and the histological response to CT. Maintenance chemotherapy consisted of vincristine + actinomycin and cytoxan + doxorubicin. The total duration of therapy was 10 months. *Results* After a median follow-up of 8.5 years, the projected overall survival at 5 years was 66% and disease-free survival (DFS) was 58%. In patients treated by surgery, only the histological response to CT had an influence on survival: 75% DFS for patients with a good histological response (less than 5% of cells), 48% for intermediate responders and only 20% for poor responders (≥ 30% of cells), *P* < 0.0001. The initial tumor volume by itself had no influence on DFS in these patients. In contrast, the tumour volume had a strong impact on DFS in patients treated by radiation therapy alone. Age had no impact on outcome. *Conclusion* Therapeutic trials for localized Ewing's sarcoma should be based on the histological response to chemotherapy or on the tumour volume according to the modality used for local therapy. © 2001 Cancer Research Campaign http://www.bjcancer.com


*British Journal of Cancer* (2001) **85**(11), 1646–1654
© 2001 Cancer Research Campaign
doi: 10.1054/ bjoc.2001.2150, available online at http://www.idealibrary.com on IDEAL

http://www.bjcancer.com

### Prognostic factors in localized Ewing's tumours and peripheral neuroectodermal tumours: the third study of the French Society of Paediatric Oncology (EW88 study)

**O Oberlin[1], MC Le Deley[2], B N'Guyen Bui[3], JC Gentet[4], T Philip[5], P Terrier[6], C Carrie[7], F Mechinaud[8], C Schmitt[9], A Babin-Boillettot[10] and J Michon[11]**

[1]Pédiatrie Institut Gustave Roussy, 39 rue Camille Desmoulins, 94805 Villejuif Cedex; [2]Département de statistiques Institut Gustave Roussy, 39 rue Camille Desmoulins, 94805 Villejuif Cedex; [3]Oncologie, Fondation Bergonié, 180 Route de Saint Genès, 33076 Bordeaux Cedex; [4]Oncologie, Hopital d'Enfants de La Timone, Avenue Jean Moulin, 13385 Marseille Cedex; [5]Pédiatrie Centre Léon Bérard, 28 rue Laennec 69008 Lyon Cedex 08; [6]Anatomie Pathologique Institut Gustave Roussy, 39 rue Camille Desmoulins, 94805 Villejuif Cedex; [7]Radiothérapie, Centre Léon Bérard, 28 rue Laennec 69373 Lyon Cedex 08; [8]Oncologie Pédiatrique CHR, Quai Moncousu, 44035 Nantes Cedex; [9]Médecine Infantile Hopital d'Enfants, Rue du Morvan, 54511 Vandoeuvre Cedex; [10]Onco-Hématologie, Hopital de Hautepierre, Avenue Molière, 67098 Strasbourg Cedex; and [11]Pédiatrie Institut Curie, 26 rue d'Ulm, 75231 Paris Cedex 05, France

**Summary** *Purpose*: (1) To improve survival rates in patients with Ewing's sarcoma (ES) or peripheral neuroectodermal tumours (PNET) using semi-continuous chemotherapy and aiming to peform surgery in all; (2) To identify early prognostic factors to tailor therapy for future studies. *Patients and methods* One hundred and forty-one patients were entered onto the trial between January 1988 and December 1991. Induction therapy consisted of five courses of Cytoxan, 150 mg/m$^2$ × 7 days, followed by Doxorubicin, 35 mg/m$^2$ i.v on day 8 given at short intervals. Surgery was recommended whenever possible. The delivery of radiation therapy was based on the quality of resection and the histological response to CT. Maintenance chemotherapy consisted of vincristine + actinomycin and cytoxan + doxorubicin. The total duration of therapy was 10 months. *Results* After a median follow-up of 8.5 years, the projected overall survival at 5 years was 66% and disease-free survival (DFS) was 58%. In patients treated by surgery, only the histological response to CT had an influence on survival: 75% DFS for patients with a good histological response (less than 5% of cells), 48% for intermediate responders and only 20% for poor responders (≥ 30% of cells), *P* < 0.0001. The initial tumor volume by itself had no influence on DFS in these patients. In contrast, the tumour volume had a strong impact on DFS in patients treated by radiation therapy alone. Age had no impact on outcome. *Conclusion* Therapeutic trials for localized Ewing's sarcoma should be based on the histological response to chemotherapy or on the tumour volume according to the modality used for local therapy. © 2001 Cancer Research Campaign http:www.bjcancer.com

**Keywords**: Ewing's tumour; chemotherapy; prognostic factors

Over the past two decades, the outcome of patients with Ewing's sarcoma has improved considerably. Most of the progress has been possible due to the development of effective chemotherapy regimens aimed at preventing distant metastases, the demonstrated cause of death in over 80% of cases. However, since the dramatic improvement experienced during the 1970s and 1980s, survival rates have plateaued, with little improvement reported beyond the 50% threshold (Nesbit et al, 1990; Wilkins et al. 1986; Jurgens et al, 1988a; Bacci et al, 1985; Barbieri et al, 1990; Kinsella et al, 1991).

In the first study of the French Society of Pediatric Oncology (SFOP), 95 patients with localized disease were initially treated, from 1978 to 1982, with combined chemotherapy comprising vincristine, dactinomycin, doxorubicin and cyclophosphamide followed by high-dose radiation therapy (45 to 60 Gy according to the location of the tumour). At 5 years, disease-free survival was 51%. In this study, surgical resection was selected for the small group of patients with expendable bones such as the ribs or the fibula (Oberlin et al, 1985).

In 1984, the SFOP initiated the EW84 study with several goals:

(1) to improve the efficacy of chemotherapy by introducing ifosfamide to replace cyclophosphamide;
(2) to obtain better local control by extending the indications for surgical resection of the primary; and
(3) to reduce therapy-induced late effects by decreasing the radiation doses in patients undergoing surgery.

This study concluded that ifosfamide did not afford a further benefit for long-term disease control compared to the previous study. However, half of the patients had undergone conservative surgery and two-thirds of them had received no or reduced doses of radiation therapy that will very likely decrease long-term sequelae (Oberlin et al, 1992).

In 1983, Hayes et al from the St Jude Children's Research Hospital in Memphis reported that previously untreated Ewing's sarcoma had achieved a dramatic response rate following moderate-dose, 2-drug, sequentially-scheduled induction chemotherapy (Hayes et al, 1983). Patients with localized or metastatic disease were given cyclophosphamide at a dose of 150 mg/m$^2$ for 7 days followed by 35 mg/m$^2$ of doxorubicin

*Received 8 May 2001*
*Revised 2 August 2001*
*Accepted 18 September 2001*

*Correspondence to:* O Oberlin on day 8. Five cycles were given before a clinical or surgical evaluation (Hayes et al, 1983). In 1989, the same team reported the overall results of this therapy for localized tumours. In terms of local therapy, only bones known to be expendable were submitted to surgery. Patients with a complete tumour resection received no irradiation. Patients with positive margins received 35–41 Gy. Patients with an unresected primary received radiotherapy, the dose and target volume being dependent on response to this induction chemotherapy: 30–35 Gy after a good response to chemotherapy or 50 Gy if there was gross residual tumour in soft tissue. Maintenance chemotherapy consisted of vincristine, dactinomycin, cyclophosphamide, and doxorubicin for a total duration of therapy of 10 months. The 5-year survival estimate for all the patients was 80%, comparing favourably with the other published series. Most of the relapses were local relapses after 35 Gy radiation (Hayes et al, 1989).

The third study, initiated by the SFOP in November 1987 had several aims:

(1) to confirm the effectiveness of such semi-continuous chemotherapy in preventing metastases;
(2) to improve the local control rate observed in the Memphis study by resecting the primary as often as possible;
(3) to reduce long-term sequelae by reducing the radiation doses and avoiding radiation therapy in selected cases; and
(4) to assess the prognostic value of factors such as the size of the primary, age, LDH, erythrocyte sedimentation rate, the histogical response to chemotherapy, neuroectodermal differentiation of the tumour.

### PATIENTS AND METHODS

### Patient selection

All patients had untreated localized small round-cell bone tumours of neuroectodermal origin, excluding lymphoma, rhabdomyosarcoma and neuroblastoma. Lesions included typical or conventional ES and atypical ES, malignant peripheral primitive neuroectodermal tumours of bone (PNET) and Askin's tumours of the thoracic wall.

There was no upper age limit prohibiting entry on the study. Informed consent was obtained from the parents or guardian of each child or from the adult patient according to the Declaration of Helsinki.

### Histopathology

The diagnosis in all cases was based on the histological analysis of biopsy samples. A panel of reference pathologists reviewed the histopathological material to confirm the diagnosis. Each diagnosis was based on the presence of small round cells occurring in the bone, with no histologic, cytologic or immunohistochemical features of lymphoma, rhabdomyosarcoma, or neuroblastoma. Tumours were classified into one of two categories: typical Ewing's sarcoma (TES) characterized by undifferentiated cells and devoid of neural markers or so-called peripheral neuroectodermal tumor (PNET) bearing neural features defined as positivity of at least one of two neural differentiation antigens (e.g., NSE or S-100 protein) by immunochemistry or the presence of Homer-Wright rosettes.

### Pretreatment evaluation

Staging procedures consisted of X-rays and computed tomography (CT) scans of the primary site and chest and a whole-body technetium bone scan. Primary tumours of the rib with even malignant pleural effusion were considered as localized disease and included in the study. Multiple aspirates and 2 biopsies were performed to assess bone marrow. The tumour volume was estimated by determining the three dimensions of the primary bone tumour and of the soft tissue mass on the pretreatment CT and the tumour volume was calculated according to the previously reported formula: volume = $a \times b \times c \times 0.52$ for 'ellipsoidal tumours' or $a \times b \times c \times 0.785$ for 'cylindrical tumours' (a: the maximal tumour dimension; b: tumour dimension perpendicular to a; c: the longitudinal maximum tumour dimension) (Gobel et al, 1987). Pretreatment chemical laboratory tests consisted of serum LDH measurement and erythrocyte sedimentation rate.

### Chemotherapy

Chemotherapy protocol was similar to that published by Hayes et al (Hayes et al, 1989). All patients received induction chemotherapy with five courses of cyclophosphamide, 150 mg/m$^2$ for 7 days followed on day 8 by doxorubicin, 35 mg/m$^2$ with courses beginning on days 1, 15, 29, 50, and 71 (Figure 1). Cyclophosphamide was given i.v. for the first course and orally for the subsequent courses, without mesna. Patients were re-evaluated on weeks 12–13 for clinical response to chemotherapy.

Radiological response assessment was based on the reduction of the soft tissue mass and graded as a complete response (complete disappearance of soft tissue mass), a partial response (more than 50% regression of soft tissue mass), no response (0–50% reduction of the soft tissue mass), progressive disease (any increase in the soft tissue mass), undulating response (early recurrence of symptoms during chemotherapy).

Local therapy was initiated on week 14. Surgical resection of the primary was recommended not only for bones known to be expendable but also for bones requiring replacement by an endoprosthesis or allograft. The surgical specimen was examined by the local pathologist to determine the histological response based on a grading system derived from Huvos's grading system for osteosarcoma (Huvos et al, 1977). Response was scored according to 3 grades: minimal residual disease (no identifiable viable tumour or less than 5% of identifiable residual tumor cells), moderate effect (5–30% of identifiable residual tumour cells) and no or small effect (more than 30% of identifiable residual tumour cells).

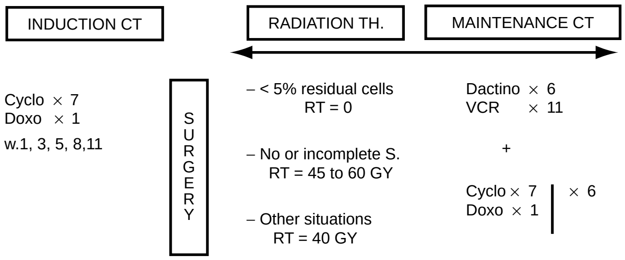

**Figure 1** Outline of the treatment schedule. CT = chemotherapy, TH = therapy, S = surgery, RT = radiation therapy, Gy = grays, Cyclo = cyclophosphamide, Doxo = doxorubicin, VCR = vincristine, Dactino = dactiomycin

© 2001 Cancer Research Campaign 

The radiation dose delivered was according to the quality of surgery and the histological response to chemotherapy. No radiation was delivered after complete resection of a tumour containing less than 5% of residual cells. In patients with an unresected or incompletely resected primary, 45 Gy were delivered to the entire tumour-bearing bone sparing one epiphyseal centre where possible. Boosts of 10–15 Gy leading to a total dose of 55 to 60 Gy were added to the bony tumour volume and the residual mass whenever possible with a 2 cm safety margin. For the other patients who had undergone complete resection but had more than 5% viable tumour cells in the specimen, the location of the tumour and the percentage of residual tumour cells were taken into account when deciding upon the radiation dose which was usually 40 Gy.

Maintenance chemotherapy was identical for all patients, whatever the response to induction chemotherapy. It was started after surgery, along with radiation therapy and consisted of weekly vincristine, 1.5 mg/m$^2$ (maximum 2 mg) for 11 weeks and dactinomycin combined with vincristine, 1.5 mg/m$^2$ (maximum 2 mg) every 2 weeks up to six doses. Subsequent therapy included six additional courses of sequential cyclophosphamide and doxorubicin at 3-week intervals. The total duration of therapy was approximately 10 months with cumulative doses of 385 mg/m$^2$ and 10.5 g/m$^2$ for doxorubicin and cyclophosphamide respectively.

### Statistical analyses

All eligible patients were included in the analysis, regardless of their compliance to the protocol. Overall, disease-free survival, local control and metastasis-free survival were calculated from the initiation of induction chemotherapy and estimated by the Kaplan-Meier method (Kaplan and Meier, 1958). Patients who failed to achieve a disease-free status, were considered as having progressive disease since the first day of therapy. All patients were included in the DFS curves. The statistical significance of prognostic variables was tested by the log-rank test (Peto et al, 1977). Multivariate analysis was then performed with the Cox proportional hazard model (BMDP program). The multivariate analysis models were determined by the likelihood ratio test.

### RESULTS

From January 1988 to December 1991, 141 patients were treated in 29 institutions in France and Belgium. Eighty-three (59%) patients were male and 58 (41%) were female. The median age was 11.9 years (range; 2–35 years). Forty-three patients (30%) were 15 years or more and 17 (12%) were 20 years or more. Eighty primary tumours (57%) were located in the trunk, 26 (18%) in proximal (humerus and femur) and the remaining 35 (25%) in distal parts of the extremities.

At diagnosis, the largest tumour dimension was 8 cm or more in 84 patients (60%). The tumour volume was less than 100 ml in 61 patients (43%), between 100 ml and 200 ml in 34 patients (24%) and greater than 200 ml in 46 patients (33%).

Table 1 details tumour characteristics according to age comparing the older group (> 15 years) to the younger one. Surprisingly, the median tumour volume was not smaller in the younger group as compared to the older group (126 ml versus 75). There was no correlation between the tumour site and age.

Ninety-two patients had an ES and 49 had a PNET. There was no difference between patients with ES or PNET in terms of gender, age, tumour site and volume.

### Clinical response to initial chemotherapy

At completion of initial chemotherapy, clinical response was assessed by imaging in 101 patients. Of these patients, 87 (85%) were considered to be good responders with a complete response in 35 patients and a partial response in 52. Fourteen patients (14%) were considered to be poor responders. There was no response in 8 children: disease progressed in 2 and an early recurrence occurred in 6 patients after an initial regression. Response could not be evaluated in 36 patients: 29 had no measurable soft-tissue mass after biopsy and 7 had undergone resection of the bone tumour at diagnosis. Four patients were not evaluated clinically before surgery.

There was no correlation between clinical response to chemotherapy and the site of the primary (trunk and extremities) nor between response and the size of the primary.

### Surgery

One hundred and eight patients (77%) underwent surgical resection of the primary tumour, either at diagnosis (7 cases) or after induction chemotherapy (101 cases). Only four patients underwent amputation because of poor response to chemotherapy in two cases and a very large tumour of the humerus and of the tibia respectively in two. One hundred and four patients had conservative surgery: either bone resection alone (45 patients) or bone resection and reconstruction (59 patients).

There was no significant correlation between the volume of the primary and the modality used for local therapy. The median volume of the resected tumours was 118 ml (range 1 to 2310 ml) versus 138 (range 3 to 1300 ml) for the unresected tumours. Surgery was performed as often in patients aged 15 years or under as in patients who were older than 15 years (Table 1) but was performed more often for extremity tumours (88%) than for trunk tumours (67% $P = 0.03$).

Histological response to induction chemotherapy was evaluated in 90 of the 101 patients who underwent surgery after induction chemotherapy. Sixty-one patients (68%) were good responders with minimal residual disease (less than 5% of residual cells); 38 of these patients had no identifiable tumour in the resected bone. Fourteen patients (15%) had an intermediate response (5–30% of residual

**Table 1** Tumour characteristics of the tumour and local therapy according to age

| | < 15 years *n* = 98 | ≥ 15 years *n* = 43 | |
|---|---|---|---|
| Volume of the primary | | | |
| median | 126 ml | 92 ml | NS* |
| range | 2–2312 ml | 4–1300 | |
| Site of the tumour | | | |
| Trunk primary | 52% | 67% | NS** |
| Limb primary | 48% | 32% | |
| Pelvic primary | (19%) | (26%) | NS** |
| Local therapy | | | |
| surgical resection ± RT | 77% | 74% | NS** |
| radiation therapy | 60% | 70% | |

*: *t*-test; ** chi square test.

*© 2001 Cancer Research Campaign*

cells) and 15 patients (17%) were poor responders, with 30% or more residual cells. Response to chemotherapy was not evaluated in 11 patients because the local pathologist lacked the expertise. The characteristics of these last tumours were not different from their evaluated counterparts in terms of site, size and outcome.

### Radiation therapy

The median dose of radiation therapy delivered to patients who did not undergo surgery (30 patients) was 60 Gy and ranged from 40 Gy (delivered to vertebral tumours) to 65 Gy.

Among the 104 patients who underwent conservative surgery, 52 did not receive additional radiation therapy, 22 received 35 to 40 Gy after complete resection of their primary and 27 received the classic high dose for positive or doubtful microscopic margins in 14, an intermediate response to chemotherapy in 3 and because the physician chose to do so in 10. Two patients developed metastasis and two had both local and distant progression before the beginning of radiation therapy.

### Overall treatment outcome

The median follow-up of the 85 survivors was 8.5 years, (range 3.5–11 years) with follow-up exceeding 7 years in 75% of these patients.

Eight patients progressed under initial chemotherapy and either did not achieve control of their primary with induction chemotherapy or developed metastases very soon after radical surgery. Fifty-five patients relapsed after initial tumour control. Seventy-eight (55%) maintained a disease-free status.

The site(s) of the first relapse was local ($n$ = 18), both local and distant ($n$ = 8), or distant alone ($n$ = 29). Among these metastases, 21 occurred in lungs, 10 in bones, 1 in bone and bone marrow, 4 in lungs and bones and 1 in lungs, bones and bone marrow. Relapses occurred at times ranging from 2 months to 102 months from the diagnosis (mean, 27 months). Forty-three (78%) of the relapses occurred within 3 years from the diagnosis. However, 4 were observed beyond 5 years (at 69, 73, 96 and 102 months from diagnosis).

The projected overall survival (OS) at 5 years was 66% (se 4) and disease-free survival (DFS) was 58% (se 4) (Figure 2).

### Prognostic factors

Table 2 lists disease-free survival rates according to the parameters investigated for their prognostic significance for DFS. No correlation was observed between these variables and the prognosis (disease-free and overall survival) in terms of gender or age at diagnosis. Figure 3 shows the 5-year DFS of patients younger than 15 years compared to that of older patients. The results would be similar in terms of overall survival or if the cut-off age was 20 years.

DFS did not vary significantly according to the site of the primary tumour. Patients with distal extremity disease fared best with 5-year DFS attaining 65% but this rate was not significantly

**Table 2** Five year disease-free survival by prognostic variables, univariate analysis

| Variables | No. | Failure | 5-year DFS (%) | Standard error | *P* |
|---|---|---|---|---|---|
| Gender | | | | | |
| male | 83 | 38 | 56 | 5 | NS (0.7) |
| female | 58 | 25 | 60 | 6 | |
| Age | | | | | |
| < 15 years | 98 | 41 | 60 | 5 | NS (0.5) |
| ≥ 15 years | 43 | 22 | 53 | 7 | |
| < 20 years | 125 | 56 | 57 | 4 | NS (0.7) |
| ≥ 20 years | 16 | 7 | 62 | 12 | |
| Site | | | | | |
| extremity | 61 | 24 | 64 | 5 | NS (0.3) |
| trunk | 80 | 39 | 54 | 4 | |
| distal extremity | 35 | 12 | 65 | 8 | NS (0.4) |
| proximal extremity | 26 | 12 | 58 | 10 | |
| trunk | 80 | 39 | 54 | 5.5 | |
| non-pelvic | 111 | 49 | 58 | 5 | NS (0.6) |
| pelvic | 30 | 14 | 57 | 10 | |
| Histo | | | | | |
| ES[1] | 92 | 43 | 55 | 5 | NS (0.6) |
| PNET[2] | 49 | 20 | 63 | 7 | |
| LDH | | | | | |
| < 500 U/L | 73 | 29 | 63 | 5.5 | NS (0.07) |
| > 500 U/L | 23 | 13 | 43 | 10 | |
| ESR | | | | | |
| < 30 | 61 | 23 | 67 | 6 | $P = 0.04$ |
| > 30 | 36 | 20 | 44 | 7 | |
| Size | | | | | |
| < 8 cm | 57 | 17 | 72 | 6 | $P = 0.002$ |
| ≥ 8 cm | 84 | 46 | 49 | 5 | |
| < 100 ml | 61 | 19 | 69 | 4 | $P = 0.006$ |
| 100–200 ml | 34 | 17 | 55 | 8 | |
| ≥ 100 ml | 46 | 27 | 45 | 7 | |

[1]: TES: Typical Ewing's sarcoma; [2]: PNET peripheral neuroectodermal tumour.

*© 2001 Cancer Research Campaign*

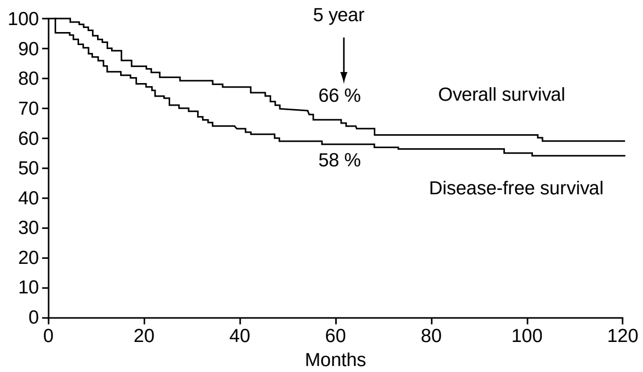

**Figure 2** Overall and disease-free survival rates ($n$ = 141)

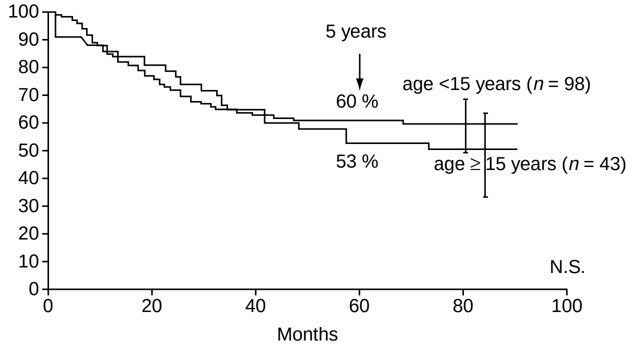

**Figure 3** Disease-free survival rates for patients younger than 15 years ($n$ = 98) and older than 15 years ($n$ = 43)

better than that of patients with proximal or axial tumours. DFS was also similar for patients with pelvic disease as compared to patients with non pelvic primary. There was no difference in outcome between patients with tumours classified as ES or PNET.

Initial pretreatment serum LDH value had no significant impact on DFS ($P$ = 0.07), whatever the chosen cut-off point, either 460 UI/L or 500 U/L. Erythrocyte sedimentation rate (ESR) at diagnosis was predictive of outcome. The probability of DFS was 63% for patients with a normal ESR and only 43% for patients with elevated ESR ($P$ = 0.04).

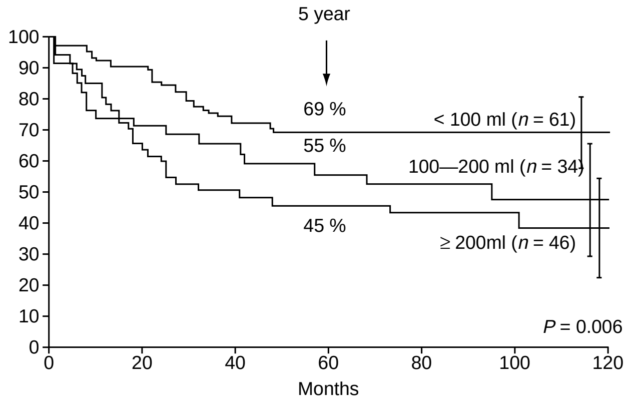

**Figure 4** Disease-free survival rates for patients with a primary tumour < 100 ml ($n$ = 61), between 100 ml and 200 ml ($n$ = 34) and ≥ 200 ml ($n$ = 46)

The relationship between the size of the primary and outcome was investigated taking into account the largest tumour dimension as well as the volume. Figure 4 shows that DFS decreased according to the size of the primary at diagnosis. DFS at 5 years was 69% for patients whose primary was < 100 ml, 55% for patients with a primary larger than 100 ml but smaller than 200 ml and only 45% for patients whose primary was ≥ 200 ml ($P$ = 0.006). Similar figures were obtained when the largest dimension was taken into account (Table 2). Analysis of the pattern of failures according to the size of the primary showed that the actuarial risk of local failure was significantly higher for large tumours (> 100 ml) than for the small tumours (< 100 ml) (30% versus 12% respectively), with the risk of metastases being similar for both groups (22% versus 25% respectively).

Volume of the tumour and ESR were strongly correlated and ESR had no more impact on survival after adjustment on tumour volume whereas tumour volume retains its prognostic value after adjustment on ESR.

DFS was correlated with clinical response to chemotherapy. None of the patients who had a clinical poor response to chemotherapy are disease-free survivors, whereas the DFS was 60% for patients who had good response. The DFS of the 36 patients who could not be evaluated because of an initial surgery or the absence of measurable soft tissue was 76%, not significantly different from the DFS of the good clinical responders ($P$ = 0.08).

**Table 3** Five-year disease-free survival by prognostic variables in patients treated by surgery ± radiation therapy, univariate analysis

| Variables | No. | Failure | 5-year DFS (%) | Standard Error | *P* |
|---|---|---|---|---|---|
| All patients | 108 | 44 | 63 | 5 | |
| Site | | | | | |
| extremity | 54 | 20 | 66 | 6 | |
| trunk | 54 | 24 | 59 | 7 | NS (0.53) |
| Volume | | | | | |
| < 100 ml | 47 | 16 | 66 | 7 | |
| 100–200 ml | 27 | 12 | 63 | 9 | |
| > 200 ml | 34 | 16 | 58 | 8 | NS (0.43) |
| Radiological response | | | | | |
| Good response | 69 | 25 | 65 | 8 | |
| Poor response | 7 | 7 | 0 | | $< 10^{-6}$ |
| Histological response | | | | | |
| Good | 61 | 16 | 75 | 5.5 | |
| Intermediate | 14 | 8 | 40 | 13 | |
| Poor | 15 | 13 | 20 | 10 | $< 10^{-6}$ |

 *© 2001 Cancer Research Campaign*

The modality used for local therapy had a significant impact on the prognosis. The 108 patients who underwent surgery fared significantly better than the 33 patients who did not. DFS at 5 years was 63% (se 5) for the former compared to only 42% (se 8) ($P = 0.04$) for the latter.

In the sub-group of 108 patients who underwent surgery (Table 3), the size of the tumour had no impact on DFS. Among this sub-group, the DFS of the 47 patients with a small primary (< 100 ml) was 66% (se 7) versus 60% (se 6) for the 61 patients with a larger tumour (≥ 100 ml). In contrast, there was a significant difference in DFS according to the size of the primary in the sub-group of 33 patients treated with radiation therapy alone: 79% (se 10) for small tumours versus only 13% (se 13) for large tumours ($P = 0.001$).

Figure 5 displays DFS curves according to the histological response to chemotherapy. At 5 years, the DFS was 75% (se 5) for the 61 patients with a good response to chemotherapy (less than 5% of residual cells), 48% (se 13) for the 14 patients with an intermediate response (more than 5% and less than 30% residual cells) and only 20% (se 10) for the 15 poor responders (≥ 30 residual cells). This difference was highly significant $P < 0.0001$.

Figure 6 shows the DFS curves for patients according to the histological response to chemotherapy and the volume of the primary. Patients with a good response to chemotherapy have a 62% DFS whatever the volume of the primary contrasting with the low DFS of patients with a poor response, in whom there was no impact of the primary tumour volume ($P < 0.0001$).

A multivariate analysis was performed for the 90 patents for whom the histological response to chemotherapy had been evaluated. As shown in Table 4, only the histological response was statistically related to disease-free survival. We did not test the impact of clinical response on DFS since it was closely related with the histological response: all the clinically poor responders were also histological poor responders.

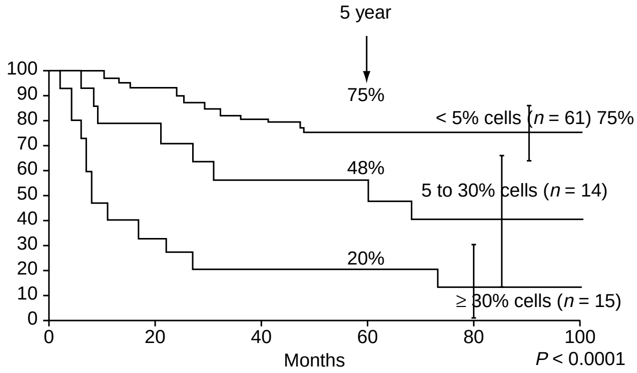

**Figure 5** Disease-free survival according to histological response to chemotherapy: Good response (< 5% cells; *n* = 61), intermediate response (5–30% cells, *n* = 14), poor response (≥ 30% cells, *n* = 15)

**Table 4** Cox's regression model for patients who had histological grading of the resected tumour after initial chemotherapy (disease-free survival)

| | Relative risk | Confidence interval 95% | P |
|---|---|---|---|
| Site | | | |
| extremity | 1 | | |
| trunk 1.1 | 0.6–2.2 | NS (0.78) | |
| Volume | | | |
| < 100 ml | 1 | | |
| 100–200 ml | 0.95 | 0.4–2.2 | |
| > 200 ml | 0.93 | 0.4–2.2 | NS (0.99) |
| Histological response | | | |
| < 5% cells | 1 | | |
| > 5% cells | 5 | 2.5–10 | $< 10^{-5}$ |

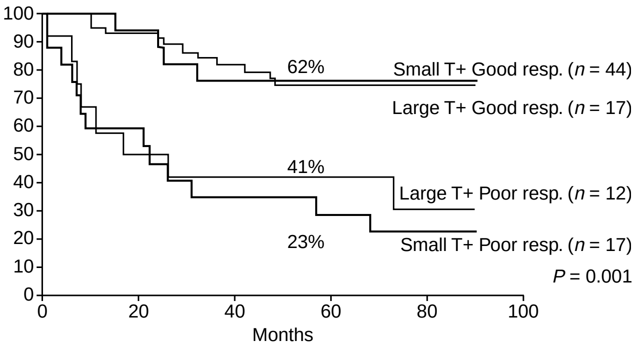

**Figure 6** Disease-free survival rates according to histological response to chemotherapy primary (good response : < 5% cells, poor response: ≥ 5% cells) and tumour volume (small tumours: < 200 ml, large tumours: ≥ 200 ml)

### Prognostic groups

As the size of the primary was only predictive of outcome in patients who did not undergo surgery and the histological response to chemotherapy was the only prognostic factor in patients who had surgery, these two criteria could be used to define two risk groups with a totally different outcome, as shown in Figure 7. The standard-risk group consists of 82 patients who either demonstrated a good response to chemotherapy – less than 5% of residual cells – ($n = 61$) or did not undergo surgery and had a small tumour at diagnosis – < 200 ml – ($n = 21$) with a DFS of 72% at 5 years (se 5). The high-risk group consists of 48 patients who either demonstrated a poor histological response – 5% of residual cells or more – ($n = 29$) or did not undergo surgery but had a large tumour at diagnosis – 200 ml or more – ($n = 12$). At 5 years, DFS was 26% for these patients (se 7).

### Toxicity

The protocol was fairly well tolerated. Chemotherapy courses did not require hospitalization. There were no therapy-related deaths. During the actinomycin + vincristine phase of chemotherapy, two patients developed veno-occlusive disease which was life-threatening in one case but the girl recovered without sequelae. During the same phase, nine patients experienced severe weight loss due to significant anorexia. The weekly vincristine (up to 11 weeks) led to neuropathy in nine patients with abdominal pain in four and limb-muscle pain in five. Seven patients developed haematuria. Two episodes occurred during induction chemotherapy and resolved with increased hydration; three occurred during maintenance cyclophophamide chemotherapy with courses one, four and six; two occurred after the end of treatment in patients who had received irradiation to the pelvis. Only one of these episodes led to the discontinuation of cyclophosphamide. Six patients had asymptomatic echocardiography abnormalities that were mild and transient.

The tolerance of radiation therapy in combination with actinomycin induced acute radiation reactions including six cases of mucositis or diarrhea and nine cases of skin reactions.

Thus far, four patients have developed a second primary. One girl who was HIV-positive developed acute lymphoblastic leukaemia 18

*© 2001 Cancer Research Campaign*

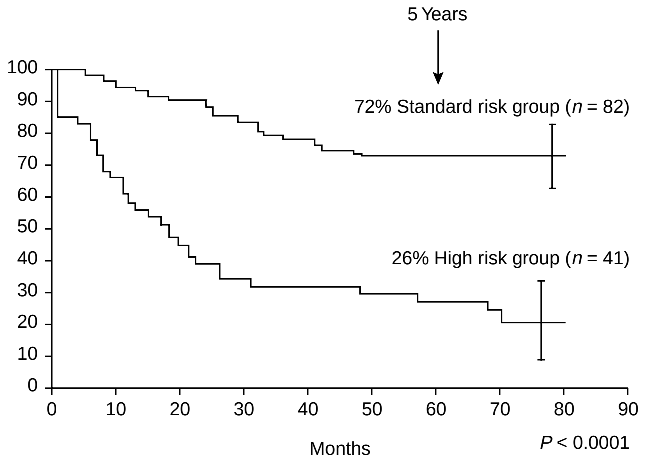

**Figure 7** Disease-free survival rates according to risk groups. (1) Standard-risk group: tumours treated by surgery after initial chemotherapy, with less than 5% of residual cells and small tumours (< 200 ml) treated by radiation therapy alone. (2) High-risk group: tumours treated by surgery with ≥ 5% of residual cells and large tumors (> 200 ml) treated by radiation therapy alone months after the end of treatment and died. One boy developed hemispheric glioblastoma 3 years after treatment of a rib primary and was alive without disease 4 years later. A 30-year-old woman developed an ovarian carcinoma 5 years after the diagnosis of Ewing's sarcoma of the fibula. An 11-year-old girl with a Ewing's tumour of the ileum developed an osteosarcoma 8 years later within the radiation field that had received a dose of 60 Gy. The cumulative incidence of second cancers is 3% (se 1.5) and 5% (se 2.5) at 5 and 7 years after diagnosis.

### DISCUSSION

When we planned the EW88 study, we were aware of the preliminary results of the protocol used by Hayes and colleagues in Memphis to treat localized Ewing's sarcoma. They had demonstrated efficient prevention of metastases in their series; only 3 of 50 patients had developed isolated metastatic relapses (Hayes et al, 1989).

The aims of the EW88 study was to achieve a better overall outcome by improving local control using a more surgery-oriented approach than that used in the Memphis study.

The overall disease-free survival of 58% at 5 years observed in the present study was similar to that observed in the above-mentioned study. Arai updated the results of that study and reported a 5-year relapse-free survival rate of 59% (Arai et al, 1991). These results also closely parallel the DFS reported by the German Co-operative Ewing's Sarcoma Study group (Hense et al, 1999) and compare favourably with the Paediatric Oncology Group's study which reported an EFS of 51% (Donaldson et al, 1998). As compared to the therapy used by the German group (Hense et al, 1999), the chemotherapy was rather mild but tightly scheduled focusing more on dose escalation in individual courses.

There have been conflicting results in the literature concerning the impact of neural differentiation on the prognosis. The results of our study do not demonstrate a relationship between histological features and the prognosis and do not support the concept that tumours with more extensive neural differentiation may carry a poorer prognosis. Bacci et al. recently found that EFS and OS rates were significantly lower in patients with PNET than that observed in patients with undifferentiated tumours (Bacci et al, 2000). Results previously reported by Terrier et al, Jurgens et al, and Parham et al, showed no differences in outcome according to neurodifferentiation (Jurgens et al, 1993; Parham et al, 1999; Terrier et al, 1995).

The tumour size appears to be a significant factor both for local tumour control and survival. This was first identified by Mendenhall et al who reported a DFS of 87% in patients whose primary lesions were grossly confined to bone compared to 20% for those with extraosseous extension. The decrease in survival related to soft tissue extension was due to an increase in distant metastasis as well as local failure, and was independent of the site of the primary (Mendenhall et al, 1983). The Memphis study confirmed a significant correlation between the size of the primary tumour and event-free survival (81% vs 49% for tumours < 8 cm vs ≥ 8 cm) (Arai et al, 1991).

The CESS81 study investigated the tumour volume and confirmed that this parameter correlates strongly with 3-year DFS (65% vs 32% for tumours < 100 ml vs ≥ 100 ml) (Sauer et al, 1987). In the CESS86 study, the treatment intensity was adapted to the tumour volume and the results suggested a shift toward a greater volume of 200 ml and above (Hense et al, 1999).

The fact that there was no correlation between tumour volume and the modality of local therapy is to be underlined and suggests the advantage of the use of surgery compared to radiotherapy in large tumours.

Patients with pelvic primary had a 55% DFS and there was no significant difference in outcome by tumour site whether lesions were subdivided into two sites (pelvic and non-pelvic sites) or three sites (central, proximal extremity, distal extremity). This is at variance with the IESS-1 experience where patients with pelvic disease had a worse survival than those with involvement of non-pelvic sites (Nesbit et al, 1990), and with the POG study which observed only a 24% EFS for patients with pelvic primary as compared to 65% for patients with distal extremity and central disease (Donaldson et al, 1998).

The impact of age at onset of disease has been debated. Several studies report a more favourable prognosis for younger patients (Bacci et al, 2000; Craft et al, 1997; Kinsella et al, 1991). In our study, tumour characteristics (site and volume), the modality used for local therapy and outcome were similar in younger and older patients. This corroborates the experience of the CESS 86 study (Hense et al, 1999).

The correlation between histologically-proven tumour necrosis after preoperative chemotherapy and clinical outcome is well-established in patients with osteosarcoma (Glasser et al, 1992). There were doubts as to whether the same grading system would be appropriate for Ewing's sarcoma as this tumour shrinks with preoperative chemotherapy and does not produce a similar osseous and cartilaginous framework facilitating the estimation of tumour necrosis based on the number of viable cells per unit area. However in Ewing's tumours, even after substantial shrinkage of the soft-tissue tumour with preoperative chemotherapy, the delineation of the initial tumour in the bone and the residual soft-tissue are identifiable. Thus the value of extrapolation of the osteosarcoma grading system was relevant. In the previous French cooperative study EW84, this grading system was used in 31 patients who underwent surgery after induction chemotherapy. All the 9 patients who had more than 50% of residual cells relapsed as compared with only 4 of the 22 patients who had less than 50% of residual cells ($P < 0.001$) (Oberlin et al, 1992). Owing to considerable

*© 2001 Cancer Research Campaign*

changes in the chemotherapy regimens used in the EW84 study and in the EW88 study we had to confirm that the histological response remained prognostic. Furthermore, in the EW88 study, as postoperative chemotherapy was modified compared to that administered in the induction regimen by the introduction of dactinomycin and vincristine, the value of the histological response to cyclophosphamide and doxorubicin also warranted documentation. We confirmed in 90 patients that this response was strongly correlated with outcome and was independent of the size of the primary at the time of the diagnosis.

The histological response of the primary was first analysed in the CESS81 study where the Salzer-Kuntschick's grading system was applied using a 10% cut-off. A large difference was found between good responders (patients exhibiting less than 10% of viable tumour) and poor responders. Of 38 good responders only 8 relapsed, whereas 11 of 16 poor responders did ($P < 0.001$). The DFS at 3 years was 79% for good responders and 31% for poor responders ($P < 0.001$). In that study, the multivariate analysis showed that the influence of the histological response appeared to have an edge over that of the tumour volume (Jurgens et al, 1988b).

The Bologna team investigated a three-grade system based on the absence of identifiable cells, the presence of microscopic or of macroscopic nodules. The 5-year DFS estimates were, respectively, 95%, 68%, and 34% (Picci et al, 1993). The same team confirmed the absence of a correlation between the tumour volume at diagnosis and chemotherapy-induced necrosis and that when the histological response to chemotherapy was taken into account, the tumour size was no longer statistically correlated with DFS as in our present experience (Bacci et al, 2000).

The prognostic factors found in this study (the histological response to chemotherapy in patients who underwent surgery and the size of the tumour in patients who did not) were the bases of the following French EW93 study which was carried out from 1993 to 1999. In that study, chemotherapy was intensified in the high-risk group. The randomized EuroEwing99 study which started a few months ago compares the role of high-dose chemotherapy with peripheral blood stem cell support to conventional chemotherapy in this high-risk group of patients. This study will also evaluate the value of an intensive up-front chemotherapy based on ifosfamide, etoposide, vincristine and doxorubicin.

### ACKNOWLEDGEMENTS

This study was supported, in part, by the Association de Recherche contre le Cancer.

We thank Ms Lorna Saint Ange for editing the manuscript.

We would like to thank all the participating centres for their contribution in the conduct of this study:

| | | |
|---|---|---|
| Angers, F | Centre Paul Papin | Dr Pein |
| Besançon, F | Hopital Saint Jacques | Dr Plouvier |
| Bordeaux, F | Hopital Pellegrin | Dr Perel |
| Bordeaux, F | Fondation Bergonié | Dr Bui |
| Bruxelles, B: | Hopital Reine Fabiola | Dr Devalck, Dr Sariban |
| Caen, F: | Hopital Côte de Nacre | Dr Boutard |
| Clermont Ferrand, | Hotel Dieu | Pr Demeocq |
| Dijon | Hopital d'enfants | Dr Couillaut |
| Grenoble, F: | Hopital A.Michalon | Dr Plantaz |
| Lille, F: | Hopital Calmette | Dr Nelken |
| Lille, F: | Centre Oscar Lambret | Dr Demaille |
| Limoges, F: | Hopital Dupuytren | Pr de Lumley |
| Lyon, F: | Centre Léon Bérard | Dr Frappaz, Pr Philip, |
| Marseille, F: | Hopital de la Timone | Dr Coze, Dr Gentet |
| Montpellier, F: | Hopital Saint Charles | Dr Marguerite |
| Nancy, F: | Hopital d'Enfants | Pr Sommelet, Dr Schmitt |
| Nantes, F: | Centre hospitalier régional | Dr Mechinaud-Lacroix |
| Nice, F: | Centre Antoine Lacassagne | Dr Thyss |
| Poitiers, F | Hopital Jean Bernard | Dr Millot |
| Paris, F: | Institut Curie | Dr Michon, Pr Zucker, Dr Pacquement, Dr Doz, Dr.Quintana |
| Paris, F | Hopital Trousseau | Pr Leverger, Pr Landmann-Parker |
| Reims, F: | Hopital américain | Dr Behar, Dr Munzer |
| Rennes, F: | Hopital Sud | Dr Bergeron, Pr Legall |
| Saint Etienne, F: | Hopital Saint Priest | Pr Freycon |
| Strasbourg, F: | Hospices Civils | Pr Lutz, Dr Babin Boilletot |
| Toulouse, F: | Centre Claudius Régaud | Dr Roché |
| Toulouse, F: | Hopital Purpan | Dr Robert, Dr Rubie |
| Tours, F: | Hopital Clocheville | Pr Lamagnère, Dr Lejars |
| Villejuif, F: | Institut Gustave Roussy | Dr Oberlin, Dr Hartmann Dr Patte, Dr Brugieres, Dr Kalifa, Dr Valteau |

### REFERENCES

Arai Y (1991) Ewing's sarcoma: local tumor control and patterns of failure following limited-volume radiation therapy. *Int J Radiat Oncol Bio Phys* **21**: 1501–1508

Bacci G (2000) Prognostic factors in nonmetastatic Ewing's sarcoma of bone treated with adjuvant chemotherapy: analysis of 359 patients at the Istituto Ortopedico Rizzoli. *J Clin Oncol* **18**: 4–11

Bacci G (1985) Localized Ewing's sarcoma of bone: ten years' experience at the Istituto Ortopedico Rizzoli in 124 cases treated with multimodal therapy. *Eur J Cancer Clin Oncol* **21**: 163–173

Bacci G (2000) Neoadjuvant chemotherapy for Ewing's sarcoma of bone in patients older than thirty-nine years [In Process Citation]. *Acta Oncol* **39**: 111–116

Barbieri E (1990) Combined therapy of localized Ewing's sarcoma of bone: analysis of results in 100 patients. *Int J Radiat Oncol Biol Phys* **19**: 1165–1170

Craft AW (1997) Long-term results from the first UKCCSG Ewing's Tumour Study (ET-1). United Kingdom Children's Cancer Study Group (UKCCSG) and the Medical Research Council Bone Sarcoma Working Party. *Eur J Cancer* **33**: 1061–1069

Donaldson SS (1998) A multidisciplinary study investigating radiotherapy in Ewing's sarcoma: end results of POG #8346. Pediatric Oncology Group. *Int J Radiat Oncol Biol Phys* **42**: 125–135

*© 2001 Cancer Research Campaign*

Glasser DB (1992) Survival, prognosis, and therapeutic response in osteogenic sarcoma. The Memorial Hospital experience. *Cancer* **69**: 698–708

Gobel V (1987) Prognostic significance of tumor volume in localized Ewing's sarcoma of bone in children and adolescents. *J Cancer Res Clin Oncol* **113**: 187–191

Hayes FA (1983) The response of Ewing's sarcoma to sequential cyclophosphamide and adriamycin induction therapy. *J Clin Oncol* **1**: 45–51

Hayes FA (1989) Therapy for localized Ewing's sarcoma of bone. *J Clin Oncol* **7**: 208–213

Hense HW (1999) Factors associated with tumor volume and primary metastases in Ewing tumors: results from the (EI)CESS studies. *Ann Oncol* **10**: 1073–1077

Huvos AG, Rosen G and Marcove RC (1977) Primary osteogenic sarcoma: pathologic aspects in 20 patients after treatment with chemotherapy en bloc resection, and prosthetic bone replacement. *Arch Pathol Lab Med* **101**: 14–18

Jurgens H (1988a) [The German Society of Pediatric Oncology Cooperative Ewing Sarcoma Studies CESS 81/86: report after 6 1/2 years]. [German]. *Klinische Padiatrie* **200**: 243–252

Jurgens H (1988b) Multidisciplinary treatment of primary Ewing's sarcoma of bone. A 6-year experience of a European Cooperative Trial. *Cancer* **61**: 23–32

Jurgens H, Paulussen M and Roessner A (1993) Neural differenciation in small cell sarcomas of bone in children and adolescents: Implications for treatment? *Proc Am Soc Clin Oncol* **12**: 1415(Abstract)

Kaplan ES and Meier P (1958) Non-parametric estimation from incomplete observation. *J Am Stat Assoc* **53**: 457–480

Kinsella TJ (1991) Long-term follow-up of Ewing's sarcoma of bone treated with combined modality therapy [see comments]. *Int J Radiat Onc Biol Phy* **20**: 389–395

Mendenhall CM (1983) The prognostic significance of soft tissue extension in Ewing's sarcoma. *Cancer* **51**: 913–917

Nesbit ME (1990) Multimodal therapy for the management of primary, nonmetastatic Ewing's sarcoma of bone: a long-term follow-up of the First Intergroup study. *J Clin Oncol* **8**: 1664–1674

Oberlin O (1985) The response to initial chemotherapy as a prognostic factor in localized Ewing's sarcoma. *Eur J Cancer Clin Oncol* **21**: 463–467

Oberlin O (1992) No benefit of ifosfamide in Ewing's sarcoma: a nonrandomized study of the French Society of Pediatric Oncology. *J Clin Oncol* **10**: 1407–1412

Parham DM (1999) Neuroectodermal differentiation in Ewing's sarcoma family of tumors does not predict tumor behavior. *Hum Pathol* **30**: 911–918

Peto R (1977) Design and analysis of randomized clinical trials requiring prolonged observation of each patient. II. analysis and examples. *Br J Cancer* **35**: 1–39

Picci P (1993) Prognostic significance of histopathologic response to chemotherapy in nonmetastatic Ewing's sarcoma of the extremities. *J Clin Oncol* **11**:1763–1769

Sauer R (1987) Prognostic factors in the treatment of Ewing's sarcoma. The Ewing's Sarcoma Study Group of the German Society of Paediatric Oncology CESS 81. *Radiother Oncol* **10**: 101–110

Terrier P (1995) Is neuro-ectodermal differentiation of Ewing's sarcoma of bone associated with an unfavourable prognosis? *Eur J Cancer* **31A**: 307–314

Wilkins RM (1986) Ewing's sarcoma of bone. Experience with 140 patients. *Cancer* **58**: 2551–2555

*© 2001 Cancer Research Campaign*
